# PpiA, a Surface PPIase of the Cyclophilin Family in *Lactococcus lactis*

**DOI:** 10.1371/journal.pone.0033516

**Published:** 2012-03-19

**Authors:** Nicolas Trémillon, Eric Morello, Daniel Llull, Rabia Mazmouz, Jean-Jacques Gratadoux, Alain Guillot, Marie-Pierre Chapot-Chartier, Laura Monlezun, Véronique Solé, Hervé Ginisty, Isabelle Poquet

**Affiliations:** 1 INRA, UMR1319 Micalis (Microbiologie de l'Alimentation au service de la Santé), Domaine de Vilvert, Jouy-en-Josas, France; 2 GTP Technology, Prologue Biotech, Labège, France; University of Kansas Medical Center, United States of America

## Abstract

**Background:**

Protein folding in the envelope is a crucial limiting step of protein export and secretion. In order to better understand this process in *Lactococcus lactis*, a lactic acid bacterium, genes encoding putative exported folding factors like Peptidyl Prolyl Isomerases (PPIases) were searched for in lactococcal genomes.

**Results:**

In *L. lactis*, a new putative membrane PPIase of the cyclophilin subfamily, PpiA, was identified and characterized. *ppiA* gene was found to be constitutively expressed under normal and stress (heat shock, H_2_O_2_) conditions. Under normal conditions, PpiA protein was synthesized and released from intact cells by an exogenously added protease, showing that it was exposed at the cell surface. No obvious phenotype could be associated to a *ppiA* mutant strain under several laboratory conditions including stress conditions, except a very low sensitivity to H_2_O_2_. Induction of a *ppiA* copy provided *in trans* had no effect i) on the thermosensitivity of an mutant strain deficient for the lactococcal surface protease HtrA and ii) on the secretion and stability on four exported proteins (a highly degraded hybrid protein and three heterologous secreted proteins) in an otherwise wild-type strain background. However, a recombinant soluble form of PpiA that had been produced and secreted in *L. lactis* and purified from a culture supernatant displayed both PPIase and chaperone activities.

**Conclusions:**

Although *L. lactis* PpiA, a protein produced and exposed at the cell surface under normal conditions, displayed a very moderate role *in vivo*, it was found, as a recombinant soluble form, to be endowed with folding activities *in vitro*.

## Introduction

During protein folding, the *cis-trans* isomerization of proline peptide bonds is a particularly slow and rate-limiting reaction catalyzed by ubiquitous Peptidyl-Prolyl *cis-trans* Isomerases (PPIases, EC 5.2.1.8) in both eucaryotes and prokaryotes [1]. PPIases belong to three families: i) Cyclophilins, ii) FK506-Binding Proteins (FKBP), and iii) parvulins [1]. i) Cyclophilins and ii) FKBP were the first described PPIase classes, and they differ by their sensitivity to immunosuppressant drugs: they are respectively inhibited by cyclosporine A or FK506 [1]. iii) Parvulins were more recently identified as PPIases, and they are specifically inhibited by juglone [2].

In the bacterial envelope, PPIases play important and diverse biological functions. In *Escherichia coli*, SurA protein, of the parvulin family, is necessary for outer membrane integrity and resistance to various stress agents (whose entry is limited by the outer membrane), because it is involved in outer membrane protein biogenesis, and even in organelle assembly [3], [4], [5], [6], [7]. In *Bacillus subtilis*, PrsA lipoprotein, another member of the parvulin family, is an essential and abundant protein that is involved in a late stage of protein secretion and required for cell morphology [8], [9] through an effect on penicillin binding protein (PBP) stability [10]. In pathogenic species, members of all three PPIase families, parvulins like SurA [7], [11] (and references therein) and PrsA homologs [12], FKBPs like MIP (Macrophage Infectivity Potentiator) proteins [13], [14], and cyclophilins like SlrA of *Streptococcus pneumoniae* [12], are involved in virulence or colonization, probably indirectly *via* the folding of exported virulence or invasion factors. However, no function could be attributed to some exported PPIases, like *E. coli* PpiA, a periplasmic cyclophilin [15].

Strikingly, some proteins assigned, by similarity, as “PPIases”, fail to display any PPIase activity *in vitro*, like the PrsA homolog (PpmA) of *S. pneumoniae* [12]. Even when PPIase activity has been established *in vitro*, it can be dispensable *in vivo*, as shown for *E. coli* SurA [16] and *B. subtilis* PrsA [17]. In the case of SurA, a demonstrated chaperone activity could be responsible for its *in vivo* function [16]. However, no chaperone activity of WT (lipomodified) PrsA protein could be evidenced *in vitro*, so the molecular mechanism underlying its action in the cell remains unknown [17].

In the biotechnology field, envelope PPIase proteins can be overproduced to improve protein production in recombinant microbial cell factories. In *B. subtilis*, PrsA is involved in the late stages of heterologous protein secretion, in particular at high levels, by favoring protein folding and/or limiting protein degradation after membrane translocation [8], [9], and PrsA overproduction can enhance the secretion of heterologous proteins, like amylases [8], [9], [18], . In *E. coli* periplasm, overproduced FkpA (FKBP family) increases the production of exported antibody fragments [20].

*Lactococcus lactis* is a gram-positive, lactic acid bacterium primarily used in the dairy industry, but also as a host to produce and secrete proteins for various biotechnological, food or medical applications [21], [22]. In this context, understanding protein quality control mechanisms in *L. lactis* is of interest [23]. *L. lactis* is characterized by a small genome, and, compared to *B. subtilis*, by relatively simple protein quality control machineries in the envelope. i) For protein degradation, lactococcal laboratory strains have a unique extra-cytoplasmic protease, HtrA [24], [25], [26]. Single *htrA* mutants lead to complete protein stability, without suffering any major growth defect under normal conditions [25], [26], [27], [28], [29], [30], and they improve secretion efficiency and yield [25], [26], [27], in contrast to *B. subtilis* regulatory mutants affected in *htrAB* expression [19]. *L. lactis htrA* mutant strains have thus widely been used as hosts to produce and secrete heterologous or recombinant proteins [28], [29], [31], [32], [33], [34], [35], [36], [37]. ii) For protein folding, two PPIases are known in *L. lactis* envelope. PrtM is a chaperone specific for envelope proteinase PrtP and it is encoded, together with its target, on plasmids specific for milk-growing strains [38], [39]. PrtM and *B. subtilis* PrsA are among the founder members of the PPIase parvulin family [2]. The PrsA homolog in *L. lactis*, PmpA, is a lipoprotein dispensable under normal conditions, in contrast to PrsA, but required for saline stress resistance [40]. PmpA is able, when slightly over-produced, to protect a heterologous secreted protein from the extra-cellular degradation [40] by HtrA protease [25], [26].

In this study, a new putative exported PPIase of the cyclophilin family, PpiA, was identified in *L. lactis* and characterized. *ppiA* expression and PpiA location were examined, and the phenotypes of inactivation and over-expression mutants were analysed *in vivo*. A recombinant secreted form of PpiA was also produced in *L. lactis* and purified from the culture medium, and its activities were assayed *in vitro*.

## Results and Discussion

### *L. lactis* PpiA is a putative exported cyclophilin

Two exported PPIases have previously been described in *L. lactis*: plasmid-encoded PrtM [38], [39] and genome-encoded PmpA [40]. In *L. lactis* genomes, there are, apart from PmpA, three other putative PPIases: two in the cytoplasm (Trigger Factor, FKBP-type, and PpiB, cyclophilin-type), and one in the envelope, PpiA (CAL96990.1 in strain MG1363 and AAK04463.1 in strain IL1403, both sharing 87% identity over their entire length).

**Figure 1:**
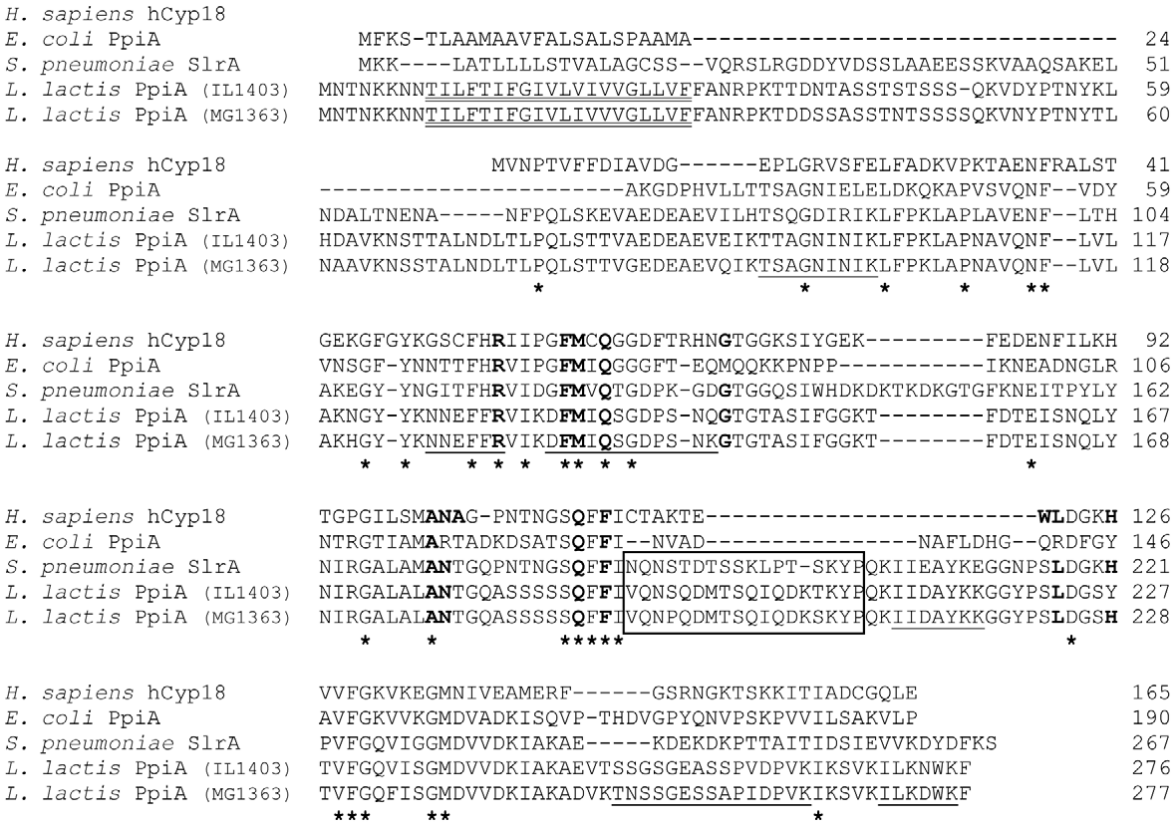
Protein sequence alignment between lactococcal PpiA protein and related cyclophilins. The sequences of some cyclophilins: *Homo sapiens* hCyp18 (Accession n° P62937), *E. coli* PpiA (P0AFL3), *S. pneumoniae* SlrA (NP_358273), and *L. lactis* PpiA (from strain IL1403: NP_266521.1 or strain MG1363: YP_001031737.1), are shown. Sequence alignment was performed using MultiAlin and manually improved to align the N-terminal hydrophobic domains of the three bacterial exported PPIases, and to take into account a previously published alignment [12]. Identical amino acids are marked with asterisks, and gaps with dash characters. The amino acids of the catalytic center of hCyp18 are marked in bold (in hCyp18 and all the proteins where they are conserved). The insertion sequence that is specific for *S. pneumoniae* SlrA compared to *E. coli* PpiA and hCyp18 [12], and conserved in *L. lactis* PpiA, is boxed. The N-terminal hydrophobic sequence of lactococcal PpiA proteins is double underlined. PpiA peptides released by shaving treatment of MG1363 cells are underlined.

PpiA is a putative cytoplasmic membrane protein that belongs to the cyclophilin family, in contrast to PmpA and PrtM, both lipoproteins of the parvulin family [40], [41]. PpiA has an N-terminal uncleavable hydrophobic domain (http://www.cbs.dtu.dk/services/TMHMM/) and is predicted to be an N-in C-out transmembrane protein (http://bioweb.pasteur.fr/seqanal/interfaces/toppred.html). It shares 31% identity with the cyclophilin prototype, the human cytosolic hCyp18 protein (also called cyclophilin A) [42], and it bears a well-conserved catalytic sequence (Figure 1). It is also homologous to *E. coli* periplasmic PpiA protein (35% identity) [15] and, to an even greater extent (51% identity), to *S. pneumoniae* SlrA lipoprotein [12]. Furthermore, both lactococcal (PpiA) and streptococcal (SlrA) cyclophilins are predicted to be cell-surface exposed (although the former is entirely embedded into the cytoplasmic membrane whereas the latter is anchored to it), and they share a 18 residue stretch of unknown function that characterizes SlrA protein when compared to other cyclophilins (*E. coli* PpiA and hCyp18) [12] (Figure 1). Interestingly, a cyclophilin homologous to SlrA and PpiA is missing in *B. subtilis*, which has two parvulin members (PrsA and YacD; data not shown).

### *ppiA* gene is constitutively expressed and PpiA protein is produced under normal conditions

Northern blotting experiments were performed in an MG1363 strain carrying an empty expression vector under normal growth conditions (at 30°C in rich medium). They revealed two mRNA whose sizes were close to each other and close to the expected size (data not shown) according to the presence of a Rho-independent terminator at the 3′ end of the gene (http://bonsai.ims.u-tokyo.ac.jp/~mdehoon/terminators/NC_009004.trms). *ppiA* gene thus seemed to be a monocistronic transcription unit either having two transcription starts (two degenerated promoters can be predicted upstream of chromosomal *ppiA* ORF, data not shown) or undergoing mRNA processing. Western Blotting experiments were then performed using antibodies against a tagged and soluble protein produced and purified in *E. coli* cells, HFFT-PpiA_Δ1–30_ (a fusion between HFFT tag, combining both His_6_ and Flag tags, and PpiA_Δ1–30_, a truncated PpiA protein devoid of its N-terminal transmembrane domain, data not shown). A protein of expected size was found to be produced, under normal growth conditions, by a control strain (Figure S1) in contrast to the isogenic *ppiA* mutant (see below).

Interestingly, after a thermal upshift, a stress condition reported in *E. coli* to induce genes for exported folding factors, and in particular exported PPIases like FkpA and SurA [43], none of the two *ppiA* mRNAs was regulated (neither up- nor down-regulated; data not shown). Genome-wide transcriptional analyses respectively showed that *ppiA* gene expression was altered neither by heat shock (confirming Northern blot results) nor by H_2_O_2_ addition (E. Guedon, personal communication). Finally, taken together, these results indicated that *ppiA* gene was constitutively expressed and that PpiA protein was synthesized under normal conditions.

### PpiA protein is exposed at the cell surface

As a putative N-in C-out transmembrane protein, the bulk of PpiA, corresponding to the enzymatic domain, should be exposed at the cell surface. To determine the PpiA location, its susceptibility to an exogenously added protease was tested. MG1363 cells from an exponential phase culture under normal conditions (at 30°C in rich medium) were treated with trypsin, as described previously for *Streptococcus pyogenes* [44], [45]. This shaving treatment did not result in detectable lysis, as no nucleic acids could be detected in the supernatant after trypsin treatment (data not shown): it thus appeared to be appropriate to specifically release surface-exposed proteins. Indeed, in all experiments described here and below, 70% (+/−10%) of the detected tryptic peptides (when excluding from the calculation peptides from ribosomal proteins which are classically detected in bacterial surface proteomes [44], [45], [46]) were from proteins predicted to be exported (secreted or surface-exposed) by SurfG+ tool [47]. In the surface proteome released from MG1363 cells by trypsin shaving, six peptides of PpiA protein, all from the predicted extracellular C-terminal part, could be detected (Table 1), and one of them is in a highly conserved region corresponding to hCyp18 catalytic center (Figure 1). Parallel shaving experiments were performed on recombinant MG1363 cells producing heterologous exported proteins (from plasmids) and grown under normal conditions, and in ten out of eleven of these experiments, PpiA peptides could also be released from the cells (data not shown). Taken together, these results in MG1363 background (MG1363 carrying expression plasmids or not) showed that PpiA was accessible to exogeneously added trypsin protease in the absence of detectable cell lysis. Thus PpiA was confirmed to be produced under normal growth conditions and shown to be an easily detected extra-cellular protein, with its putative catalytic region exposed at the cell surface.

**Table 1:** PpiA peptides released by shaving treatment of lactococcal cells.

Accession Number	Gene name/Protein function	*E*-value (Protein)	Coverage	Identified peptides	*E*-value (Peptide)
125623254	*ppiA*/Peptidyl-prolyl *cis-trans* isomerase	6.3 10^−36^	19%	D_135_ **FM**I**Q**SGDPSNK_146_ T_251_NSSGESSAPIDPVK_265_ T_94_SAGNINIK_102_ N_126_NEFFR_131_ I_212_IDAYKK_218_ I_271_LKDWK_276_	6.9 10^−8^ 2.3 10^−7^ 5.3 10^−5^ 7.5 10^−3^ 1.2 10^−2^ 4.5 10^−2^

Six peptides identified by LCMS/MS were found to match with the same protein: its accession number, the gene name and protein function, *E*-values (for the whole protein and for each peptide) and coverage are indicated. In the first peptide, the amino acids in bold are conserved between *L. lactis* PpiA and hCyp18, and in the latter, they belong to the active center (see Figure 1).

### *ppiA* gene is dispensable under normal and stress conditions

*L. lactis* MG1363 *ppiA* gene could be inactivated to create a *ppiA* mutant strain (Figure S1). *ppiA* gene was thus found not to be essential under laboratory conditions, like *pmpA* gene from *L. lactis* [40]. Several phenotypes: colony and cell morphology, growth, and sensitivity to several stresses on plates, i. e. high temperature, NaCl, to which a *pmpA* mutant is sensitive [40], puromycin (allowing to prematurely release newly synthesized peptides from the ribosome) or lysozyme (a cell wall stress), were then examined in both *ppiA* mutant and control strains. No phenotype related to the absence of *ppiA* gene could be detected (data not shown). When exponential cells were exposed to H_2_O_2_ and their viability was measured, a low sensitivity of the *ppiA* mutant (less than one log) could be observed (data not shown). Finally, essentially no role could be assigned to *L. lactis* PpiA surface protein, with exception of a modest role in H_2_O_2_ resistance. Such a situation is not unprecedented: other PPIases have previously been shown to play a limited role, if any, in the cell under laboratory conditions. This is also the case for representatives of the three families as in *E. coli*, all four PPIases are dispensable [6], and in particular for cyclophilins: i) in *E. coli*, PpiA periplasmic cyclophilin is not essential and no phenotype of the mutant could be detected [15], and ii) in *S. pneumoniae*, SlrA is a dispensable protein under both normal and stressful laboratory conditions [12].

### Effect of *ppiA* over-expression on cell growth and viability

To study the effect of PpiA overproduction, a regulated *ppiA* copy was provided *in trans* from a plasmid: the entire *ppiA* ORF from the IL1403 strain was cloned into a previously described expression vector [48] under the control of P_Zn_, a promoter regulated by ZitR and inducible by EDTA addition [48], [49], leading to pVE8077. In an otherwise WT background (strain MG1363), *ppiA* over-expression (from pVE8077) after EDTA addition, had no effect on growth (data not shown).

The effect of *ppiA* overproduction was also tested in an *htrA* mutant background, because of previous results obtained in *Saccharomyces cerevisiae*. In this species, a gene for a nucleolar PPIase (FKBP family), *frp3*, was isolated as a partial multi-copy suppressor of the thermosensitivity of a *htrA* (*ynm3*) mutant strain, and found, when over-expressed in this *ynm3* mutant, to be able to restore a low level of thermoresistance, i. e. one log improvement [50]. Lactococcal MG1363Δ*htrA* strain [25] was transformed by pVE8077 or the empty vector, and recombinant cells were grown at high temperature (37°C and 39°C) after addition of EDTA to induce P_Zn_-controlled expression. The induction of the *ppiA* copy provided *in trans* had no significant effect on the *htrA* mutant growth or viability (data not shown). Thus lactococcal PpiA, when overproduced, was unable to rescue the growth defect of the *htrA* mutant strain at high temperature.

### Effect of *ppiA* over-expression on exported proteins

As a model for an exported protein misfolded *in vivo*, we used Exp5-Δ_SP_Nuc, an exported fusion to the staphylococcal nuclease (Nuc) reporter [51] that is highly degraded by HtrA surface protease [26]. Irrespective of the presence of overproduced PpiA, the same degradation pattern of Exp5-Δ_SP_Nuc was observed in all extracts, without any difference in total or extra-cellular protein amounts (Figure S2). Overproduced PpiA protein was thus unable to protect Exp5-Δ_SP_Nuc from proteolysis by HtrA. Interestingly, the other lactococcal PPIase, PmpA, when overproduced, was shown to be able to protect an heterologous secreted protein, the *Staphylococcus hyicus* lipase, from being degraded [40] by HtrA protease [25], [26]. Several hypotheses could account for the unability of PpiA to counteract HtrA for the degradation of Exp5-Δ_SP_Nuc: PpiA could be inactive, poorly efficient or active only under specific conditions or on specific substrates (see below).

PpiA was also found to have no effect on the secretion of several heterologous proteins (data not shown), thus suggesting that it could be of limited interest, if any, to improve protein secretion in *L. lactis*. A similar finding has previously been reported for another exported cyclophilin: in *E. coli*, endogenous PpiA, when overproduced, was found to have no role in the periplasmic production of antibody fragments, except in one case [52]. Moreover, in *B. subtilis*, even PrsA parvulin, the PPIase best known to improve heterologous secretion, was revealed to have a much more limited role than previously thought: PrsA was found to have no effect on nine out of eleven industrially interesting heterologous proteins, and thus to display a quite narrow specificity, in particular for heterologous alpha-amylases [19], its first identified substrates [8], [9].

### Purified rPpiA protein, a recombinant secreted form of PpiA, shows chaperone activity

As in a number of cases, PPIase activity had been observed *in vitro* even in the absence of *in vivo* phenotype, a recombinant PpiA protein was designed to be purified and used in activity assays. A recombinant soluble form of PpiA (rPpiA) was produced and secreted in *L. lactis*. A 5′-truncated *ppiA* ORF from strain IL1403, encoding PpiA_Δ1–30_ (a PpiA protein deleted of its N-terminal part including the transmembrane domain), was cloned into an expression-secretion vector to be fused to a lactococcal signal peptide (SP_Exp4_) ORF [25], [53] and expressed, as a gene fusion, under the control of EDTA-inducible P_Zn_ promoter [48], [49]. After growth and induction of the resulting strain MG1363(pGTP_FZ301_PpiA), rPpiA protein was found, as expected, to be secreted into the culture medium, at a yield of about 40 mg/L (data not shown). rPpiA was then purified from the culture supernatant by Ion Exchange Chromatography and size exclusion chromatography. As a control, a recombinant soluble PmpA protein, rPmpA, was also produced, secreted and purified in *L. lactis* using the same procedure as rPpiA (data not shown).

**Figure 2:**
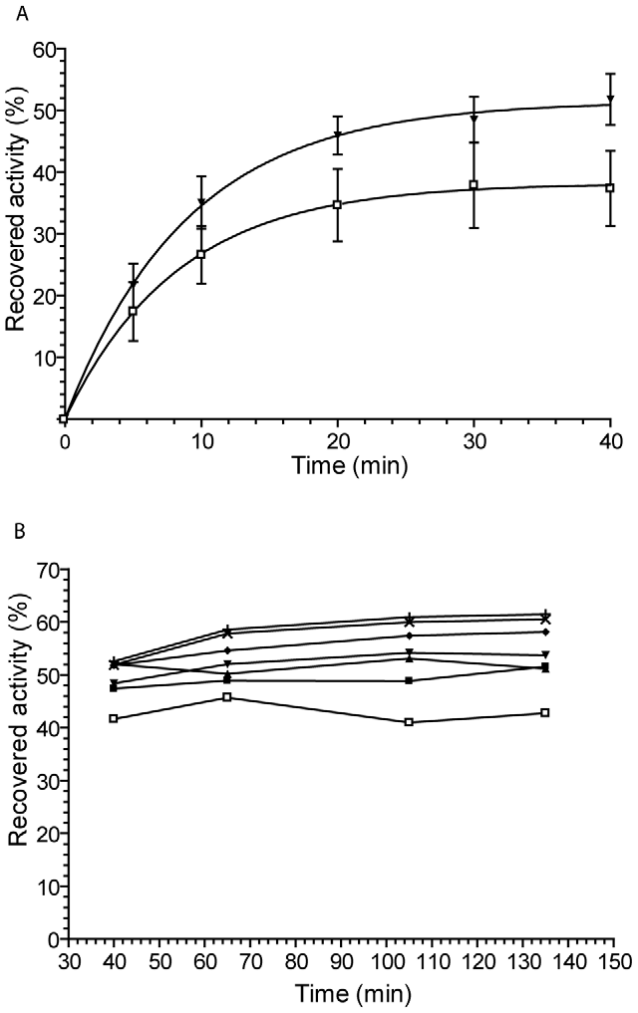
Chaperone activity of rPpiA. **A.** Citrate synthase (CS) was treated by concentrated guanidinium hydrochloride, and reactivation of unfolded CS was initiated by a 100-fold dilution into a buffer in the absence (□) or presence of rPpiA added at a CS∶rPpiA ratio of 1∶2 (▾). CS enzymatic activity was measured at the indicated time points, and recovered activity is shown (activity of native CS alone at the same concentration was set to 100%). **B.** CS refolding was followed like in A, in the absence (□) or presence of rPpiA at the following CS∶rPpiA ratios: 2∶1 (▪), 1∶1 (▴), 1∶2 (▾), 1∶5 (⧫), 1∶10 (×) and 1∶20 (+).

Chaperone activity was then assayed *in vitro* using porcine heart Citrate Synthase (CS) as a substrate (Figure 2). CS, initially unfolded, is diluted in the presence or not of a putative chaperone, and refolding is followed by the kinetics of CS activation [54]. Activity recovery after dilution is calculated as a percentage of native CS activity that is measured by a colorimetric assay. Preliminary experiments performed with HFFT-PpiA_Δ1–30_, the soluble tagged protein produced in *E. coli*, failed to reveal any chaperone activity (data not shown), suggesting that the HFFT tag (37 amino acids) could impair the activity or folding of PpiA extra-cytoplasmic domain. The activity of rPpiA and rPmpA, the soluble untagged proteins produced in *L. lactis*, were subsequently tested. In a first set of experiments, recombinant PPIases and CS were mixed at a unique stoichiometric ratio (CS∶rPPIase) of 1∶2. CS alone was, as expected, able to recover 35–40% of its activity (Figure 2A). In the presence of rPmpA, only a small improvement could be detected (data not shown). However, a chaperone activity of WT PmpA protein, as previously proposed [40], could not be excluded from those experiments, as the structure and/or activity of recombinant soluble rPmpA protein could have been affected compared to that of the WT lipo-modified PmpA protein. In the presence of rPpiA, CS activation was significantly increased by about 15% to reach 50% of native activity (Figure 2A), indicating that rPpiA displayed a moderate chaperone activity. In a second set of experiments, only the maximum reactivation of CS after prolonged time reactions and using different CS∶rPpiA ratios ranging from 2∶1 to 1∶20, was measured (Figure 2B). CS activity recovery, was increased, from the 40% level reached by CS alone, to 50% in the presence of rPpiA at CS∶rPpiA ratios as little as 2∶1 and 1∶1, thus confirming that rPpiA displayed a true, although moderate, chaperone activity. CS activity recovery was further increased as a function of rPpiA concentration, probably revealing some non-specific activity of rPpiA when provided in high amounts. Finally, these results demonstrated that purified rPpiA, when provided in low amounts (close to stoichiometric amounts), was able, in contrast to HFFT-PpiA_Δ1–30_, to exhibit chaperone activity on CS refolding.

### rPpiA protein shows Peptidyl-Prolyl Isomerase activity

**Figure 3:**
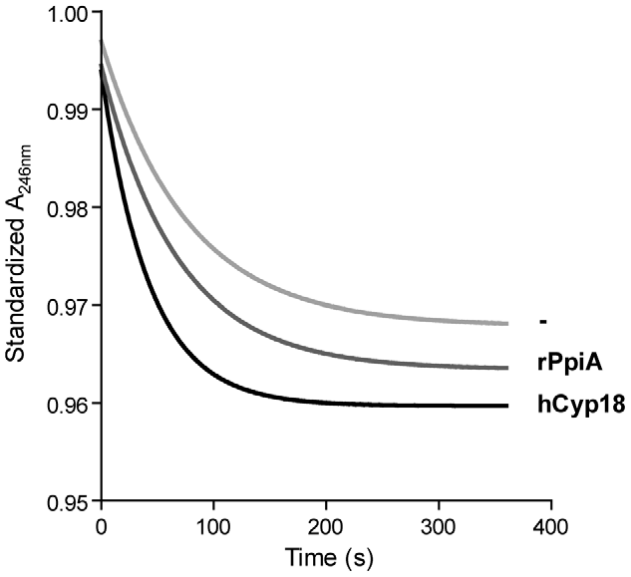
Isomerization activity of rPpiA. A protease-free assay was used to measure PPIase activity. The prolyl *cis*→*trans* isomerisation of a tetrapeptide (Suc-Ala-Ala-Pro-Phe-2,4-difluoroanilide) was followed at 6°C by the decrease in absorbance at 246 nm (A_246 nm_) as a function of time. Effects of PPIase addition (at a final concentration of 10 nM) or not (-, light grey line) were compared, using two different PPIases: rPpiA (grey line) or, as a positive control, hCyp18 (black line). The average of three independent experiments is shown.

To test PPIase activity of rPpiA, the usual competitive, protease-coupled assay could not be used, because of rPpiA sensitivity to chymotrypsin (data not shown). An alternative, protease-free assay has been described [55]. It is based on a succinyl-tetrapeptide–difluoroanilide whose *cis* and *trans* conformers display different absorption coefficients at 246 nm, so that the peptidyl prolyl *cis*→*trans* isomerization can be followed by a decrease in absorbance at 246 nm. As expected, when Suc-Ala-Ala-Pro-Phe-2,4-difluoroanilide was diluted in the presence hCyp18, used as a positive control, absorbance decrease was accelerated (Figure 3). In contrast to purified rPmpA protein (data not shown), purified rPpiA was able, although to a lesser extent than hCyp18, to speed up the tetrapeptide *cis-trans* isomerization (Figure 3), showing that rPpiA was endowed with PPIase activity. Similar results were previously obtained in *S. pneumoniae*: in contrast to the recombinant form of PpmA protein (the parvulin homologous to both *L. lactis* PmpA and *B. subtilis* PrsA), the recombinant form of SlrA was found to display PPIase activity, although at a lower level than hCyp18 [12].

rPpiA was thus shown to have both PPIase and chaperone activities, whereas the chaperone activity of its closest homolog, SlrA, has not been tested [12]. So, even though a double activity had previously been shown for a few bacterial PPIases: a parvulin (SurA), and a FKBP protein (FkpA) in *E. coli* [16], [56], to our knowledge, *L. lactis* PpiA is the first bacterial exported cyclophilin to be endowed with both PPIase and chaperone activities.

In conclusion, our study showed that *L. lactis* PpiA was produced at an easily detectable level and exposed at the cell surface during normal growth, and that it displayed both chaperone and PPIase activities *in vitro*. However, only a modest role in stress resistance, and no role in heterologous secretion, could be evidenced *in vivo*, under the conditions we tested. To account for this discrepancy, several hypotheses can be envisioned. PpiA and another exported chaperone could be functionally redundant, as first proposed in *E. coli* [57], [58]. In *L. lactis*, the only known chaperone is PmpA [40], and a functional redundancy between PpiA and PmpA cannot be excluded. However, as PmpA, in contrast to PpiA, is able to behave as an antagonist of HtrA protease and is required for resistance to NaCl stress [40], this putative redundancy would not be entirely reciprocal. To account for these results, PpiA could be involved in the same functions as PmpA, but much less efficiently. Alternatively, PpiA could be involved in some specific function and required under specific conditions. For example, in *L. lactis*, PrtM is indirectly required for lactococcal growth on milk [38] (and reference therein) [39]. In *E. coli*, the four envelope PPIases (FkpA, a FKBP protein, PpiA, a cyclophilin, PpiD and SurA, both parvulins) which are dispensable under laboratory conditions, were proposed to have significant roles in specific environments or ecological niches [6], and SurA protein was subsequently found, in an uropathogenic (UPEC) strain, to be required *in vivo* for invasion of the host and virulence [59]. Similarly, in *S. pneumoniae*, SlrA, a dispensable protein under both normal and stressful laboratory conditions, was found to be specifically required for colonization of the host [12]. In *L. lactis*, by analogy with SlrA protein, PpiA protein could play a significant role under specific, although not yet identified, conditions. The understanding of PPIase function and their conditions of activity *in vivo* requires the knowledge of their substrates, but only very few substrates of PPIases, all of the parvulin family, are known: i) *L. lactis* PrtP, the envelope proteinase, needs PrtM to be active and able to degrade caseins, and finally to allow growth on milk [38] (and reference therein) [39]; ii) *E. coli* pilins require SurA to assemble into functional pili that are important invasion factors in virulent strains [3], [4], [5], [6], [59]; and iii) *B. subtilis* penicillin binding proteins, including the essential PBP2a protein, are dependent on PrsA for their folding, which makes PrsA itself an essential protein [10]. Whether PpiA protein contibutes to protein folding in *L. lactis* cell envelope will deserve further investigation.

## Methods

### Strains and plasmids, Growth Conditions

Bacterial strains and plasmids are described in Table 2. *E. coli* strains (New England Biolabs, Ipswich, MA) were grown at 37°C with 200–250 rpm shaking in reconstituted Luria Bertani (LB) broth: 1% tryptone (Sigma, St Louis, MO), 5% yeast extract (Fluka, St Louis, MO), 1% NaCl (Fluka), dissolved in pure water, and supplemented with ampicillin 100 µg/mL or kanamycin 50 µg/mL (Sigma) when necessary. Solid media were prepared by adding technical agar (Invitrogen, Paisley, UK) at a final concentration of 1.5% w/v. *L. lactis* strains were grown at 30°C without shaking in rich M17 (Fluka) supplemented with 1% glucose (GM17) and, when necessary, with erythromycin (5 µg/mL), chloramphenicol (5 µg/mL) or tetracycline (10 µg/mL).

**Table 2:** Strains and plasmids used in this study.

Strain or plasmid	Relevant genotype or phenotype	Reference(s)/Source
**STRAINS**		
***L. lactis***		
MG1363	*L. lactis* ssp. cremoris, plasmid free derivative of NCDO712	
*ppiA*	*ppiA* mutant of strain MG1363 obtained by Single Cross-Over insertion, MG1363 *ppiA*::pRV_ppiA, Em^R^	This work
Ctl (*ppiA^+^*)	Control *ppiA^+^* strain derived from MG1363 by Single Cross-Over insertion, MG1363 *ppiA*::pRV_ppiAT, Em^R^	This work
Δ*htrA*	*htrA* deletion mutant of MG1363 strain	[25]
***E. coli***		
TOP 10	F^−^ *mcr*A Δ(*mrr*-*hsd*RMS-*mcr*BC) Φ80*lac*Z ΔM15 Δ*lac*X74 *rec*A1 *ara*D139 Δ(*ara*-*leu*) 7697 *gal*U *gal*K *rps*L (Str^R^) *end*A1 *nup*G	Invitrogen
BL21(DE3)	B F- *dcm ompT hsdS* (r_B_- m_B_-) *gal*	Stratagene
DH5α	*fhuA2* Δ*(argF-lacZ)U169 phoA glnV44 Φ80* Δ*(lacZ)M15 gyrA96 recA1 relA1 endA1 thi-1 hsdR17*	
**PLASMIDS**		
pRV_300	*E. coli* cloning vector (pBlueScript derivative), Amp^R^ *L. lactis* suicide vector, Em^R^	[60]
pRV_ppiA	pRV_300 derivative carrying an internal fragment (502 bp) of *ppiA_MG1363_* gene used to inactivate *ppiA* gene in strain MG1363	This work
pRV_ppiAT	pRV_300 derivative carrying a 3′-fragment (889 bp) of *ppiA_MG1363_* gene used for silent insertion on MG1363 chromosome, at the 3′ end of *ppiA*	This work
pGTP_c101a	Expression vector derived from pET28 (EMD Biosciences, San Diego, CA), Kan^R^ carrying an ORF coding for HFFT tag under the control of T7 promoter	This work
pGTP_c101a_PpiA	pGTP_c101a derivative after cloning ‘*ppiA_IL1403_*, Kan^R^ coding for HFFT-PpiA_Δ1–30_ fusion	This work
pGTP_FZ301	Lactococcal secretion vector, Cm^R^ carrying an ORF for SP_Exp4_ (Exp_4_ signal peptide) under the control of P_Zn_ *zitR* expression system	[53]
pGTP_FZ301_PpiA	pGTP_FZ301 derivative after cloning ‘*ppiA_IL1403_*, Cm^R^ coding for a precursor protein (a fusion between SP_Exp4_ signal-peptide and PpiA_Δ1–30_) leading to secreted rPpiA protein	This work
pVE8062	Lactococcal expression vector carrying P_Zn_ *zitR* expression system, Em^R^	[48]
pVE8064	pVE8062 derivative, Em^R^, carrying of a recombinant reporter ORF under the control of P_Zn_ *zitR* expression system	[48]
pVE8077	pVE8064 derivative after subcloning of WT *ppiA_IL1403_* ORF instead of the reporter, Em^R^	This work
pVE8078	pVE8064 derivative after reporter deletion	This work
pCR2.1-TOPO	*E. coli* cloning vector, Kan^R^, Amp^R^	Invitrogen
pCR2.1-TOPO_ppiA	pCR2.1-TOPO derivative carrying the WT ORF *ppiA_IL1403_* (including RBS) coding for WT PpiA, Kan^R^, Amp^R^	This work
pIL2608	Lactococcal vector derived from pIL105, Tet^R^	J. Anba, unpublished
pVE8021	pFUN derivative carrying *exp5-Δnuc*, Em^R^	[51]
pVE8070	pIL2608 derivative carrying *exp5-Δnuc* from pVE8021, Tet^R^	This work

Cm^R^, Amp^R^, Em^R^, Tet^R^ and Kan^R^: chloramphenicol, ampicillin, erythromycin, tetracyclin and kanamycin resistance.

### Tryptic digestion of bacterial surface proteins

Cells from a MG1363 culture grown to an OD_600 nm_ of 0.7, were harvested by centrifugation at 2,000 g for 10 min at 4°C. Surface proteins were digested by trypsin essentially as previously described [44], with some modifications. Briefly, after washing, bacteria were resuspended in one-hundredth volume of PBS containing 40% sucrose, and trypsin (Promega, sequencing grade modified) was added at a concentration of 10 µg/mL. The mixture was incubated for 5 min at 37°C, under shaking. An aliquot of the supernatant was analyzed by electrophoresis in a 0.7% agarose gel stained with ethidium bromide to reveal nucleic acids whose presence in detectable amounts would be an indicator of bacterial lysis. The supernatant was collected after centrifugation at 20,000 g for 10 min at 4°C. Trypsin (1.6 µg/mL) was added again in the supernatant and further incubated for 2 hours at 37°C. Tryptic peptides were purified by RP-HPLC on a C18 column (Aquapore reverse phase RP300, 30×2.1 mm, 7 µm; Applied Biosystems), before analysis by nano LC-MS/MS.

### LC-MS/MS analysis and database searching

LC-MS/MS analysis was performed on a Ultimate 3000 LC system (Dionex, Voisins le Bretonneux, France) connected to a LTQ Orbitrap mass spectrometer (Thermo Fisher, USA) by nanoelectrospray ion source. Tryptic peptide mixtures (4 µL) were loaded at flow rate 20 µL/min onto precolumn Pepmap C18 (0.3×5 mm, 100 Å, 5 µm; Dionex). After 4 min, the precolumn was connected with the separating nanocolumn Pepmap C18 (0.075×15 cm, 100 Å, 3 µm) and the linear gradient was started from 2 to 36% of buffer B (0.1% formic acid, 80% ACN) in buffer A (0.1% formic acid, 2% ACN) at 300 nL/min over 50 min. Ionization was performed on liquid junction with a spray voltage of 1.3 kV applied to non-coated capillary probe (PicoTip EMITER 10 µm tip ID; New Objective, USA). Peptides ions were automatically analyzed by the data dependent method as follows: full MS scan (m/z 300–1600) on Orbitrap analyser and MS/MS on the 4 most abundant precursors on the LTQ linear ion trap. In this study only +2 and +3 charged peptides were subjected to MS/MS experiments with an exclusion window of 1.5 min, with classical peptides fragmentation parameters (Qz of 0.25, activation time of 30 ms, collision energy of 40%).

The raw data produced on LTQ-Orbitrap mass spectrometer were first converted in mzXML file with ReADW (http://sashimi.sourceforge.net) and in a second step, protein identification was performed with X!Tandem software 1 (X!Tandem tornado 2008.02.01.3; http://www.thegpm.org) against a protein database of *L. lactis* MG1363 (NCBI: NC_009004), associated to a proteomic contaminant database. The X!Tandem search parameters were trypsin specificity with one missed cleavage and variable oxydation states of methionine. The mass tolerance was fixed to 10 ppm for precursor ions and 0.5 Da for fragment ions. The final search results were filtered using a multiple threshold filter applied at the protein level and consisting of the following criteria: protein log (*E*-value)<−8 identified with a minimum of two different peptides sequences, detected with a peptide *E*-value<0.05.

### Chromosomal inactivation of *ppiA* gene

Both i) an internal fragment (502 bp) and ii) a control 3′ fragment (889 bp) of the *ppiA* gene were PCR-amplified from MG1363 genomic DNA using the following primer pairs, respectively (Table S1): i) SPF-XhoI and SPR-EcoRI-STOP, and ii) SPTF-XhoI and SPTR-EcoRI. Both fragments were digested by *Xho*I and *Eco*RI and ligated into pRV300, a suicide vector in *L. lactis* [60]. The ligation mixture was transformed into competent cells of *E. coli* TOP10 strain, and white ampicillin resistant clones were selected. The resulting plasmids: i) pRV-ppiA and ii) pRV-ppiAT were verified by restriction and sequencing, and then transformed into *L. lactis* MG1363 strain. Single cross-over insertion events were selected on GM17 agar plates supplemented with erythromycin and verified by PCR analysis. Insertion of i) pRV-ppiA and ii) pRV-ppiAT on MG1363 chromosome respectively resulted in i) the *ppiA* mutant strain, and ii) the control strain (ctl) which carries the WT *ppiA* gene and the same erythromycin resistance marker as the *ppiA* mutant so that both strains can be cultivated under the same conditions.

### PpiA overexpression in *L. lactis*

*ppiA* ORF (including RBS sequence) from IL1403 strain (GenBank Accession number AAK04463.1) was cloned on an expression vector to avoid, when overexpressed in MG1363 strain, any recombination with the chromosomal gene copy. *ppiA* ORF was PCR-amplified from IL1403 genomic DNA using ppiARBS and ppiATer primers (Table S1), and the resulting *ppiA* fragment (961 bp) was ligated into pCR2.1-TOPO (Invitrogen). The ligation was then transformed into *E. coli* DH5α competent cells. The resulting plasmid, pCR2.1-TOPO_ppiA, was verified by both restriction and sequencing. *ppiA* ORF was recovered from pCR2.1-TOPO_ppiA by *Bam*H and *Eco*RV double digestion, subcloned into pVE8064 (instead of *usp-nuc*; [48]) using the same enzymes, to be under the control of P_Zn_ *zitR* expression system. The ligation was transformed into *L. lactis* MG1363 strain, and the resulting pVE8077 plasmid was verified by restriction and sequencing. In parallel, the gene fusion encoding Exp5-Δ_SP_Nuc (in operon with an upstream gene [51]) was released from pVE8021 plasmid [51] by *Sma*I and *Spe*I digestion, and cloned into pIL2608 vector after *Sac*II digestion, filling in by T4 DNA polymerase and *Spe*I digestion.

### Resistance tests

5 µL of successive dilutions of overnight bacterial cultures were spotted on GM17-agar containing (or not) NaCl at 2.5%, puromycine at 17 µg/mL or lysozyme at 1 mg/mL, and incubated at 30°C or 37°C (for the thermo-sensitivity assay) for 24–48 hours. Growth was also followed at high temperatures (37°C and 39°C). After exposure to H_2_O_2_ (2 and 4 mM), viability (cfu) was determined on GM17-agar.

### Protein analysis

2 mL of *L. lactis* cultures at a given OD_600 nm_ were harvested by centrifugation at 4°C and 10,000 rpm. The cell pellet was washed with 1 mL TE buffer (10 mM Tris, 1 mM EDTA, pH 8.0), resuspended in 0.875 mL of TE buffer filled out with 125 µL TCA 80% (v/v) and kept on ice for 20 min. The cell pellet was harvested by centrifugation and washed with 1 mL cold acetone 80% (v/v). The cell pellet was then allowed to dry for 20 min at room temperature, after which it was resuspended in 100 µL TE per OD_600 nm_ unit containing lysozyme (10 mg/mL). After 30 min of incubation at 37°C, the cells were lysed with equal volumes of SDS 8%. Cellular extracts and untreated supernatant aliquots were analyzed by SDS-PAGE, using a 15% polyacrylamide gel in Tris-glycine buffer. The gels were stained with Coomassie blue G250, followed by gel scanning (GS800 Calibrated densitometer, Biorad). Western blotting experiments were performed using antibodies against HFFT-PpiA_Δ1–30_ (this study, see below) or against staphylococcal nuclease, Nuc (Eurogentec). Gel imaging was performed with Image Quant (Amersham Biosciences,Uppsala Sweden).

### Cloning of *ppiA* gene fragments to produce recombinant proteins

A 5′-truncated fragment of *ppiA* gene (‘*ppiA* ORF coding for PpiA_Δ1–30_, i. e. PpiA deleted for its 30 first residues including the transmembrane domain) was PCR-amplified from *L. lactis* IL1403 genomic DNA (to avoid recombination with the MG1363 gene copy) using by high fidelity Phusion™ DNA polymerase (Finnzymes, Espoo, Finland) with 196-ppiA-S and 196-ppiA-R primers (Table S1). The resulting PCR product (772 bps) was digested by *Bam*HI and *Xba*I, and then ligated into two different expression vectors: i) pGTP_FZ301 [53] and ii) pGTP_c101a, a pET28 derivative. ‘*ppiA* ORF was thus fused in frame to ORFs encoding respectively i) SP_Exp4_, a lactococcal signal peptide [25], [51], or ii) HFFT, a fusion between His_6_ and Flag tags. Ligation mixtures were respectively transformed into electro-competent cells of *L. lactis* MG1363 or *E. coli* DH5α strains. The resulting pGTP_FZ301_PpiA and pGTP_c101a_PpiA plasmids were verified by restriction and sequencing (Eurofins MWG Operon, Ebersberg, Germany).

### Production in *E. coli* and purification of a soluble tagged form of PpiA

For the production of HFFT-PpiA_Δ1–30_ protein in *E. coli*, pGTP_c101a_PpiA plasmid was first introduced into strain BL21(DE3) (Stratagene, La Jolla, CA). Strain BL21(DE3) (pGTP_c101a_PpiA) strain was grown to an OD of 0.6 and the culture was induced by 0.1 mM IPTG. After 3 hours of induction, cells were resuspended in a lysis buffer (20 mM sodium phosphate, 300 mM NaCl, 10 mM Imidazole, pH 7.5) containing a protease-inhibitor cocktail (Roche Applied Bioscience, Meylan, France), and lysed by sonication. HFFT-PpiA_Δ1–30_ was then purified from cell extracts by Immobilized Metal Affinity Chromatography followed by Ion Exchange Chromatography. The soluble cellular fraction was first loaded on a C16/20 column (GE Healthcare) packed with 10 mL Chelating Sepharose Fast Flow resin (GE Healthcare) and equilibrated in lysis buffer without imidazole (20 mM sodium phosphate, 300 mM NaCl at pH 7.5). After elution using increasing concentrations of imidazole (10 mM, 30 mM, 60 mM and 500 mM), fractions containing HFFT-PpiA_Δ1–30_ were pooled, diluted 10-fold in 20 mM TrisHCl (pH 7.5) and then purified using a Vantage 10/40 column (Millipore, Billerica, MA) packed with 10 mL Q-sepharose Fast Flow resin (GE Healthcare) and equilibrated in 20 mM TrisHCl (pH 7.5). HFFT-PpiA_Δ1–30_ was eluted with NaCl using a linear gradient of concentrations (from 0 to 1 M), and elution fractions were automatically collected using FRAC910 (GE Healthcare). Purified HFFT-PpiA_Δ1–30_ protein (pure at 95% as shown by SDS-PAGE analysis) was dialyzed in a storage buffer (50 mM Tris-HCl at pH 8.0, 100 mM NaCl, 2 mM EDTA) and stored at −20°C. A rabbit serum containing polyclonal antibodies against HFFT-PpiA_Δ1–30_ was obtained (Eurogentec, Seraing, Belgium).

### Production, secretion and purification of rPpiA protein in *L. lactis*

For rPpiA production and secretion in *L. lactis*, MG1363 (pGTP_FZ301_PpiA) strain was grown to mid-exponential phase in 500 mL of GM17 medium maintained at pH 6.5 (by adding NH_4_OH while continuously homogeneizing the medium by agitation at 100–150 rpm with a magnetic stirrer). At OD_600_ 2.5, the culture was induced by the addition of 1 mM EDTA for 5 hours. The culture supernatant was then filtered on a 0.22 µm membrane. An SDS-PAGE analysis of a supernatant aliquote revealed that, as expected, rPpiA protein was secreted into the culture medium, and the protein yield was of about 40 mg/L (data not shown). rPpiA was then purified by Ion Exchange Chromatography and size exclusion chromatography. All purification steps were performed on an AKTA purifier (GE Healthcare, Hillerod, Denmark). Filtered supernatant was loaded on an SP (SulfoPropyl) sepharose column (GE Healthcare) previously equilibrated in 20 mM sodium phosphate at pH 6.5, and rPpiA was eluted with a linear gradient of 0–1 M NaCl. Elution fractions were automatically collected into 2.5 mL fractions using FRAC910 (GE Healthcare) and analyzed by SDS-PAGE. rPpiA protein was further purified using Hiload 26/60 Superdex 75 prep grade resin (GE Healthcare) equilibrated in 20 mM sodium phosphate at pH 8. rPpiA protein was then concentrated using a Spectra POR#1 dialysis membrane (cut off at 6–8 kDa, Spectrum, Rancho Dominguez, CA) against 500 mL of a concentration buffer (50 mM Tris-HCl, 100 mM NaCl, 2 mM EDTA, 5% glycerol, 30% PEG20000, pH 8) and dialyzed against 250 mL of a dialysis buffer (50 mM Tris-HCl, 100 mM NaCl, 2 mM EDTA, pH 8). rPpiA was pure at more than 95% (data not shown). N-terminal micro-sequencing (Proteodynamics, Clermont Ferrand, France) confirmed that rPpiA N-terminal sequence was as expected after processing of the recombinant precursor (SP_Exp4_-DDTGRFGS-PpiA_Δ1–30_) and cleavage of its signal peptide (data not shown). For activity assays, purified rPpiA was concentrated and dialyzed in the appropriate activity buffer using a Centricon device (cut off threshold of 10 kDa, Millipore, Billerica, MA).

### Chaperone activity on Citrate Synthase

Citrate synthase from porcine heart (Sigma), whose activity can be measured as previously described [54], was first unfolded in a denaturation buffer (50 mM Tris HCl, 2 mM EDTA, 10 mM NaCl, 15 mM dithioerythritol, 6 M Guanidinium hydrochloride at pH 8) for 2 hours on ice. Unfolded CS was then diluted (100-fold) to a final concentration of 0.15 µM in a dilution buffer (50 mM Tris HCl, 2 mM EDTA, 10 mM NaCl, pH 8) in the presence or not of either rPpiA or rPmpA at various concentrations, and incubated at 25°C. At different time points, an aliquote (20 µL) of the refolding mix were added to 0.98 mL of an assay buffer (50 mM Tris HCl, 2 mM EDTA, 10 mM NaCl, 0.1 mM Oxalo Acetic Acid, 0.1 mM DTNB, 0.15 mM Acetyl CoA, pH 8). Absorbance at 412 nm was used to follow citrate synthase activity, and thus refolding as a function of time.

### PPIase activity

PPIase activity was determined using a previously described protease-free assay [55], and as a substrate, a tetrapeptide-difluoroanilide: Suc-Ala-Ala-Pro-Phe-2,4-difluoroanilide (Bachem AG, Bubendorf, Switzerland) in solution in trifluoroethanol containing 0.47 M LiCl at 6°C. After addition or not of rPpiA, rPmpA or, as a positive control, human cyclophilin A (Sigma), tetrapeptide-difluoroanilide *cis/trans* isomerization was followed as a function of time (t) by the decrease in absorbance at 246 nm (A_246_) every 1.5 s during 6 minutes, using a Beckman Coulter DU800 spectrophotometer (Beckman Coulter, Fullerton, United States). To facilitate curve comparison, values of A_246_ at t [A_246_ (t)] were standardized according to the following formula, where A_246 ST_ (t) and A_246 MAX_ are respectively the standardized value of A_246_ (t) and the maximal value: A_246 ST_ (t) = 1−[A_246 MAX_−A_246_ (t)].

## Supporting Information

Figure S1 ***ppiA*** **is expressed under normal conditions.** *ppiA* mutant strain (*ppiA^−^*) and its control (ctl) were grown to the exponential phase, and protein extracts were prepared. A Western blot analysis was performed using antibodies against HFFT-PpiA_Δ1–30_, a tagged and soluble protein that had been produced and purified in *E. coli*.
(TIF)

Figure S2 **Effect of overproduced PpiA on an exported and highly degraded hybrid protein.** The effect of PpiA over-production on export and degradation of a hybrid protein, Exp5-Δ_SP_Nuc [26], [51], was tested. Strains MG1363(pVE8077, pVE8070) and MG1363(pVE8062, pVE8070) both produce Exp5-Δ_SP_Nuc, in the presence of PpiA (encoded by a plasmid *ppiA* copy, +) or not (−). They were grown in rich GM17 medium to the exponential phase, and EDTA (500 µM) was added (+, to induce the expression of plasmid *ppiA* copy that is under the control of P_Zn_ promoter) or not (−). After 2 h of growth, protein extracts were prepared from cells (C) and supernatants (SN) and submitted to a Western-Blot analysis using anti-Nuc (A) or anti-HFFT-PpiA_Δ1–30_ antibodies (B). In (A), the intact cellular form and the extracellular degradation products of Exp5-Δ_SP_Nuc are indicated by arrows. In (B), on the right, only the cell extracts of the same strains as in (A) were analysed, and on the left, purified rPpiA was added as a positive control (dilution factors are indicated). Although several cellular proteins were found to be immuno-reactive both in the absence a *ppiA* plasmid copy and in the absence of induction (probably by cross-reaction), a unique band of about 34 kDa, close to PpiA predicted size, could specifically be detected in the presence of an induced *ppiA* plasmid copy, and was assigned to overproduced PpiA (arrow). In both (A) and (B), the size of molecular weight markers (MWM) is indicted on the left.
(TIF)

Table S1 **Primers used in this study.** Restriction sites are underlined and a reverse stop codon is in bold.
(DOCX)
