# Supplementary figures and images for: PpiA, a Surface PPIase of the Cyclophilin Family in Lactococcus lactis
Source: PLoS One. 2012 Mar 19;7(3):e33516. doi: 10.1371/journal.pone.0033516 (PMC3307742; doi:10.1371/journal.pone.0033516)

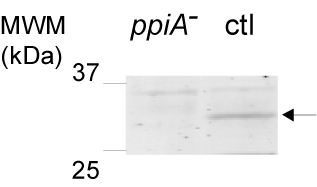

Supplement: Figure S1 — ppiA is expressed under normal conditions. ppiA mutant strain (ppiA−) and its control (ctl) were grown to the exponential phase, and protein extracts were prepared. A Western blot analysis was performed using antibodies against HFFT-PpiAΔ1–30, a tagged and soluble protein that had been produced and purified in E. coli. (TIF) [file pone.0033516.s001.tif]

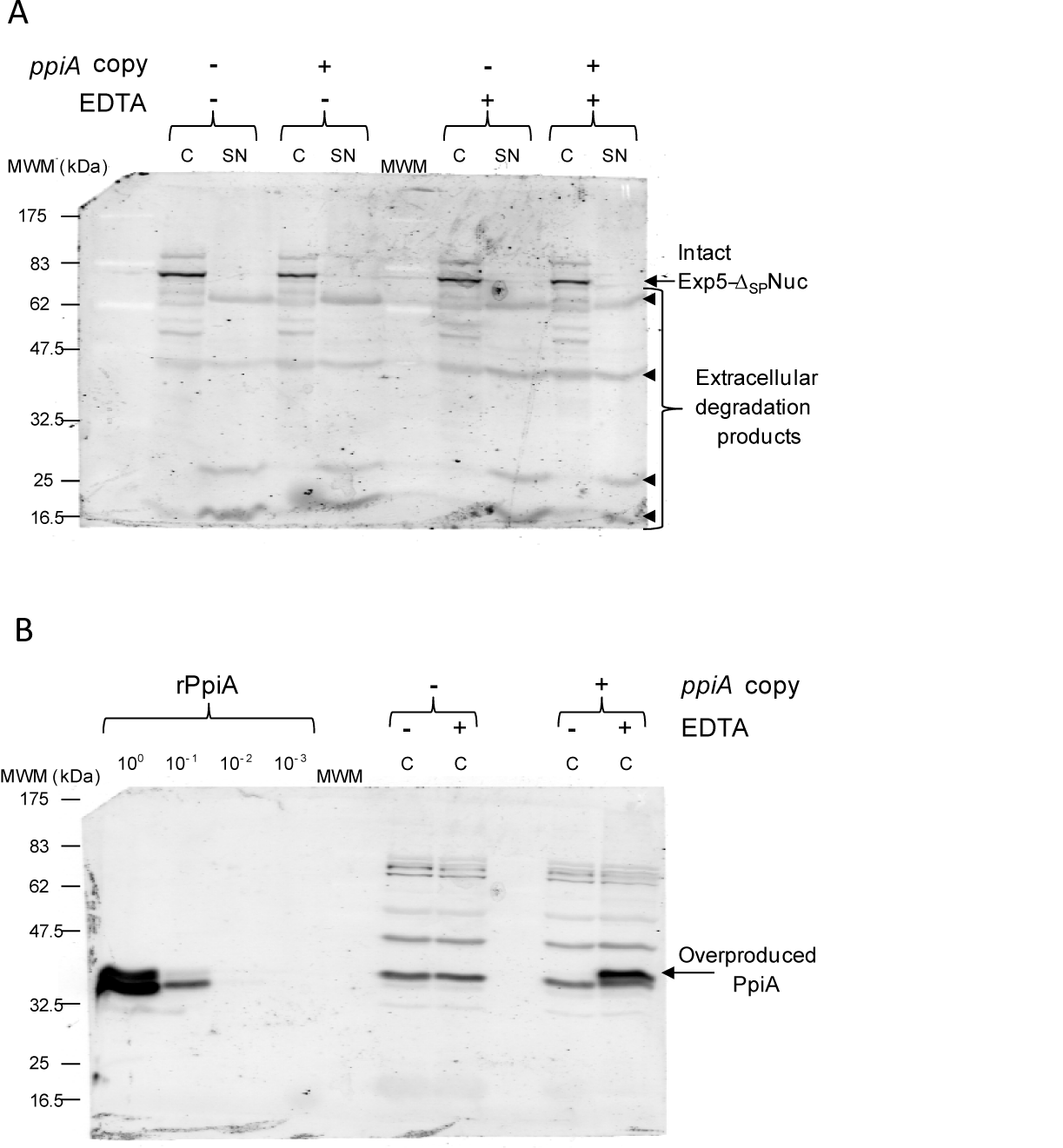

Supplement: Figure S2 — Effect of overproduced PpiA on an exported and highly degraded hybrid protein. The effect of PpiA over-production on export and degradation of a hybrid protein, Exp5-ΔSPNuc [26], [51], was tested. Strains MG1363(pVE8077, pVE8070) and MG1363(pVE8062, pVE8070) both produce Exp5-ΔSPNuc, in the presence of PpiA (encoded by a plasmid ppiA copy, +) or not (−). They were grown in rich GM17 medium to the exponential phase, and EDTA (500 µM) was added (+, to induce the expression of plasmid ppiA copy that is under the control of PZn promoter) or not (−). After 2 h of growth, protein extracts were prepared from cells (C) and supernatants (SN) and submitted to a Western-Blot analysis using anti-Nuc (A) or anti-HFFT-PpiAΔ1–30 antibodies (B). In (A), the intact cellular form and the extracellular degradation products of Exp5-ΔSPNuc are indicated by arrows. In (B), on the right, only the cell extracts of the same strains as in (A) were analysed, and on the left, purified rPpiA was added as a positive control (dilution factors are indicated). Although several cellular proteins were found to be immuno-reactive both in the absence a ppiA plasmid copy and in the absence of induction (probably by cross-reaction), a unique band of about 34 kDa, close to PpiA predicted size, could specifically be detected in the presence of an induced ppiA plasmid copy, and was assigned to overproduced PpiA (arrow). In both (A) and (B), the size of molecular weight markers (MWM) is indicted on the left. (TIF) [file pone.0033516.s002.tif]
