# Supplementary material for: PpiA, a Surface PPIase of the Cyclophilin Family in Lactococcus lactis
Source: PLoS One. 2012 Mar 19;7(3):e33516. doi: 10.1371/journal.pone.0033516 (PMC3307742; doi:10.1371/journal.pone.0033516)
Supplement: Table S1 — Primers used in this study. Restriction sites are underlined and a reverse stop codon is in bold. (DOCX) [file pone.0033516.s003.docx]

**Table S1. Primers used in this study**

Restriction sites are underlined and a reverse stop codon is in bold.

| **Name** | **Sequence** | **Restriction site** |
| --- | --- | --- |
| Rdi37-PS_Exp4_-R3 | 5’ TTTTTTGGATCCAAACCTGCCAGTATCATCAGCAAATACAACGGCT 3’ | *Bam*HI |
| Rdi37-P_Zn_-S3 | 5’ GATATATATATGGTCCAGATCTTTGATCAAGGATCTGTC 3’ | *Bgl*II |
| Rdi37-P_Zn_-RsrII-R2 | 5’ TCCTTAAGGTACGGACCGAGTATTTTGTCCTCCATAATTAGT 3’ | *Rsr*II |
| Rdi37-P_Zn_-RsrII-R | 5’ TCCTTAAGGTACGGACCGTCTTCATCGAAACTCTTCAGT 3’ | *Rsr*II |
| 196-ppiA-S | 5’ TATATATCGCGGATCCAATCGTCCAAAAACAACTGATAATAC 3’ | *Bam*HI |
| 196-ppiA-R | 5’ AATTAACCCTCTAGATTAAAATTTCCAGTTTTTAAGAATTTTAAC 3’ | *Xba*I |
| ppiARBS | 5’ AATTTATAATTACTCATTTGGATCCTAGGATATTCCTATGAATACG 3’ | *BamH*I |
| ppiATer | 5’ CGGATATCTTGAATTAGACATGCCAGAAG 3’ | *EcoR*V |
| SPR-XhoI | 5’ GGTGGCCGGCCTCGAGTTTACAATCTTTGGGATTGTCC 3’ | *Xho*I |
| SPR-EcoRI-**STOP** | 5'GCTCGTGCGCGAATTCTATTTGC**TTA**AGCAAGTGCTCC 3' | *Eco*RI |
| SPTF-XhoI | 5' GGTGGCCGGCCTCGAGCAAATTATACCTTAAATGCTGC 3’ | *Xho*I |
| SPTF-EcoRI | 5' GCTCGTGCGCGAATTCATTAGACATGCCAGATGATATG 3’ | *Eco*RI |
